# A multi-center study on low-frequency rTMS combined with intensive occupational therapy for upper limb hemiparesis in post-stroke patients

**DOI:** 10.1186/1743-0003-9-4

**Published:** 2012-01-20

**Authors:** Wataru Kakuda, Masahiro Abo, Masato Shimizu, Jinichi Sasanuma, Takatsugu Okamoto, Aki Yokoi, Kensuke Taguchi, Sugao Mitani, Hiroaki Harashima, Naoki Urushidani, Mitsuyoshi Urashima

**Affiliations:** 1 Department of Rehabilitation Medicine, The Jikei University School of Medicine, 3-25-8, Nishi-Shimbashi, Minato-Ku, Tokyo 105-8461, Japan; 2 Shimizu Hospital, 129, Miyagawa-Cho, Kurayoshi, Tottori 682-0881, Japan; 3 Department of Neurosurgery, Tokyo General Hospital, 3-15-2, Egota, Nakano-Ku, Tokyo 165-8906, Japan; 4 Nishi-Hiroshima Rehabilitation Hospital, 6-265, Miyake, Saeki-Ku, Hiroshima, Hiroshima 731-5143, Japan; 5 Department of Rehabilitation Medicine, Tokyo General Hospital, 3-15-2, Egota, Nakano-Ku, Tokyo 165-8906, Japan; 6 Division of Molecular Epidemiology, The Jikei University School of Medicine, 3-25-8, Nishi-Shimbashi, Minato-Ku, Tokyo 105-8461, Japan

**Keywords:** Repetitive transcranial magnetic stimulation, Occupational therapy, Stroke, Upper limb hemiparesis, Rehabilitation

## Abstract

**Background:**

Both low-frequency repetitive transcranial magnetic stimulation (rTMS) and intensive occupational therapy (OT) have been recently reported to be clinically beneficial for post-stroke patients with upper limb hemiparesis. Based on these reports, we developed an inpatient combination protocol of these two modalities for the treatment of such patients. The aims of this pilot study were to confirm the safety and feasibility of the protocol in a large number of patients from different institutions, and identify predictors of the clinical response to the treatment.

**Methods:**

The study subjects were 204 post-stroke patients with upper limb hemiparesis (mean age at admission 58.5 ± 13.4 years, mean time after stroke 5.0 ± 4.5 years, ± SD) from five institutions in Japan. During 15-day hospitalization, each patient received 22 treatment sessions of 20-min low-frequency rTMS and 120-min intensive OT daily. Low-frequency rTMS of 1 Hz was applied to the contralesional hemisphere over the primary motor area. The intensive OT, consisting of 60-min one-to-one training and 60-min self-exercise, was provided after the application of low-frequency rTMS. Fugl-Meyer Assessment (FMA) and Wolf Motor Function Test (WMFT) were performed serially. The physiatrists and occupational therapists involved in this study received training prior to the study to standardize the therapeutic protocol.

**Results:**

All patients completed the protocol without any adverse effects. The FMA score increased and WMFT log performance time decreased significantly at discharge, relative to the respective values at admission (change in FMA score: median at admission, 47 points; median at discharge, 51 points; p < 0.001. change in WMFT log performance time: median at admission, 3.23; median at discharge, 2.51; p < 0.001). These changes were persistently seen up to 4 weeks after discharge in 79 patients. Linear regression analysis found no significant relationship between baseline parameters and indexes of improvement in motor function.

**Conclusions:**

The 15-day inpatient rTMS plus OT protocol is a safe, feasible, and clinically useful neurorehabilitative intervention for post-stroke patients with upper limb hemiparesis. The response to the treatment was not influenced by age or time after stroke onset. The efficacy of the intervention should be confirmed in a randomized controlled study including a control group.

## Background

Application of repetitive transcranial magnetic stimulation (rTMS) influences neural excitability of selected brain areas. It has been reported that low-frequency rTMS of ≤ 1 Hz suppresses while high-frequency rTMS of ≥ 5 Hz activates local neural activities [1-4]. Several randomized controlled trials have recently confirmed that low-frequency rTMS applied to the non-lesional hemisphere can significantly improve motor function of the affected upper limb in post-stroke patients [5-7]. With regard to the underlying mechanism of the beneficial effects of rTMS, it is speculated that low-frequency rTMS to the non-lesional hemisphere reduces interhemispheric inhibition towards the lesional hemisphere, leading to facilitation of beneficial functional reorganization in the lesional hemisphere [8,9]. On the other hand, intensive occupational therapy (OT), such as constraint-induced movement therapy (CIMT) for upper limb hemiparesis has also been reported to activate peri-lesional areas in the lesional hemisphere in chronic stroke [10,11], and intensive OT has been confirmed in a study of a large number of patients to be significantly beneficial [12]. Based on this background, an inpatient combination protocol of low-frequency rTMS and intensive OT as a therapeutic approach for post-stroke patients with upper limb hemiparesis was developed at our department. In this regard, high-frequency rTMS combined with CIMT has been applied previously in this patient population at another institution [13]. In our protocol, low-frequency rTMS applied to the non-lesional hemisphere is combined with intensive OT consisting of one-to-one training and self-exercise, each provided twice per day during hospitalization [14,15]. The results of the pilot study with a small number of patients showed that the combination treatment of low-frequency rTMS and intensive OT was well tolerated by all patients. Furthermore, patients who received the treatment showed motor functional improvement of the affected upper limb. However, these previous clinical reports on low-frequency rTMS/intensive OT were based on data obtained at only one institution (Department of Rehabilitation Medicine, Jikei University School of Medicine). So far, no data about the safety, feasibility and outcome of our proposed intervention is available from other institutions. Extension of the protocol across multiple institutions is desirable, to test and confirm the feasibility and safety of the protocol in a larger heterogeneous population. The primary purpose of this multi-institutional study was to confirm the safety and feasibility of the 15-day protocol of low-frequency rTMS/intensive OT for post-stroke patients with upper limb hemiparesis. The secondary purpose was to investigate the effect of low-frequency rTMS/intensive OT on motor function of the affected upper limb in more than 200 post-stroke patients from multiple institutions. In addition, the study was also designed to identify predictors of the outcome of the intervention on motor function of the affected upper limb, although we could not include control subjects in this study based on the intervention design of the study.

## Methods

### Study Institutions

Five hospitals (Jikei University Hospital, Jikei Daisan Hospital, Shimizu Hospital, Kenkoukai Tokyo Hospital, Nishi-Hiroshima Rehabilitation Hospital) in Japan with at least 30-bed ward/section specially equipped for long-term stroke rehabilitation participated in this study (three hospitals are located in Tokyo area and two hospitals in Chugoku Area of Western Japan). All hospitals had more than or equal to two board-certificated physiatrists and more than or equal to five occupational therapists with expertise in stroke rehabilitation. For this study, the Department of Rehabilitation Medicine, Jikei University School of Medicine served as an administrative and data management center. The steering committee comprised the 15 principal investigators from these five institutions. All data were transmitted electronically to the data management center where they were analyzed. Prior to the treatment, the physiatrists and occupational therapists from each institution received a training program for standardizing the therapeutic protocol of low-frequency rTMS/intensive OT at the Department of Rehabilitation Medicine, Jikei Daisan Hospital for at least 5 consecutive days. The training program included familiarization with rTMS application, the intensive OT protocol and clinical measures used for the study.

The ethics committees of the five institutions approved the protocol of the study and intervention, and informed consent was obtained from all patients before study entry. This study was carried out in compliance with the Helsinki Declaration. All physiatrists and occupational therapists who were involved in the study were registered as NovEl Intervention Using Repetitive TMS and Intensive Occupational Therapy (NEURO) Investigators.

### Study Participants

The study subjects were 204 consecutive patients with a history of stroke and long-standing upper limb hemiparesis. They were hospitalized to one of the participating institutions for 15 days to receive low-frequency rTMS and intensive OT, between April 1, 2009 and January 31, 2011. First, all patients were referred to the outpatient clinic of the Department of Rehabilitation Medicine, Jikei University School of Medicine, Tokyo, Japan, as potential candidates. Second, after confirmation that the candidate met all the inclusion criteria set by our department for therapeutic application of low-frequency rTMS and intensive OT as described previously [14,15], each patient was assigned to one of five institutions participating in this study according to their residential address and scheduled to receive the intervention there. Briefly, the inclusion criteria were: *1) *Brunnstrom stage for hand-fingers of 3-5 (ability, at least subjectively, to flex all the fingers of the affected upper limb in full range of motion). *2) *Age at intervention between 18-90 years. *3) *Time between the onset of stroke and intervention of more than 12 months. *4) *History of a single stroke only (no bilateral cerebrovascular lesion). *5) *No cognitive impairment with a pretreatment Mini Mental State Examination score of more than 26. *6) *Clinical confirmation of the plateau state, representing no score increase in the Fugl-Meyer Assessment (FMA) by an occupational therapist from the institution in the latest 3 months. *7) *No active physical or mental illness requiring medical management. *8) *No recent history of seizure (within one year preceding the intervention). *9) *No documented epileptic discharge on pretreatment electroencephalogram. *10) *No current use of antiepileptic medications for the prevention of seizure. *11) *No pathological conditions known to be contraindications for rTMS in the guidelines suggested by Wassermann [16].

### Therapeutic Intervention

During 15-day hospitalization, each subject received 22 treatment sessions of 20-min low-frequency rTMS and 120-min intensive OT daily (two sessions per day, except for the days of admission/discharge and Sundays) (Table 1). Each OT session was scheduled to start within 10 minutes after the application of rTMS, since the room for rTMS application is close to the room used for OT (on the same floor) at each hospital.

**Table 1:** The schedule of 15-day protocol of combination treatment of low-frequency rTMS and intensive OT (example for a patient admitted on Thursday).

	Thursday	Friday-Saturday	Sunday	Monday-Saturday	Sunday	Monday-Wednesday	Thursday
Morning	Admission	Low-frequency rTMS (20 min)	No treatment	Low-frequency rTMS (20 min)	No treatment	Low-frequency rTMS (20 min)	Post-treatment evaluation
							
		One-to-one training (60 min)		One-to-one training (60 min)		One-to-one training (60 min)	
							
		Self-exercise (60 min)		Self-exercise (60 min)		Self-exercise (60 min)	

Afternoon	Pre-treatment evaluation	Low-frequency rTMS (20 min)		Low-frequency rTMS (20 min)		Low-frequency rTMS (20 min)	Discharge
							
		One-to-one training (60 min)		One-to-one training (60 min)		One-to-one training (60 min)	
							
		Self-exercise (60 min)		Self-exercise (60 min)		Self-exercise (60 min)	

Intensive OT consisted of one-to-one training and self-exercise was provided within 10 minutes after application of low-frequency rTMS to the non-lesional hemisphere.

**Figure 1:**
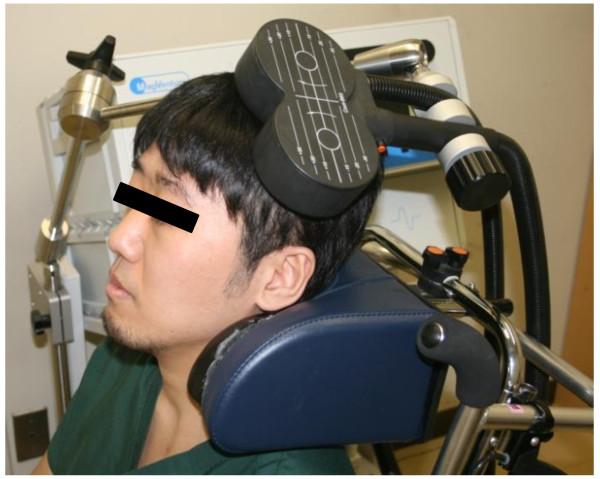
**Application of low-frequency rTMS to the non-lesional hemisphere**. Patients were seated in a chair during the application.

Low-frequency rTMS was applied using a 70-mm figure-8 coil and MagPro R30 stimulator (MagVenture Company, Farum, Denmark). According to the current safety recommendations, focal 1 Hz rTMS was applied to the contralesional hemisphere over the primary motor area. Each rTMS session consisted of 1,200 pulses, lasting 20 minutes (Figure 1). The optimal site of stimulation on the skull was defined as the location where the largest motor evoked potentials (MEPs) in the first dorsal interosseous (FDI) muscle of the unaffected upper limb was elicited on surface electromyography. The resting motor threshold (MT) of the FDI muscle of the unaffected upper limb was defined as the minimum stimulus intensity that produced a minimal motor evoked response (about 50 μV in at least 5 of 10 trials) of the muscle at rest [17,18]. According to the measured resting MT level, the intensity of stimulation was set at 90% of resting MT of the FDI muscle. For safety monitoring, a physiatrist from the institution briefly examined the patient before and after each rTMS session, paying attention to the possible development of known adverse effects of rTMS (e.g., headache, nausea, convulsion), appearance of new neurological symptoms (e.g., motor disturbance of the unaffected limbs), and deterioration of upper limb hemiparesis.

The program of intensive OT comprised two components; 60-min one-to-one training and 60-min self-exercise. The 60-min one-to-one training, which was introduced individually in a face-to-face fashion by an occupational therapist, mainly involved the application of shaping and repetitive task practice techniques. The shaping technique concentrated on the use of the affected upper limb in functional tasks chosen by each patient and the occupational therapist according to their life style and severity of hemiparesis, including reaching forward to move a cup from one place to another, wiping the surface of the table with a towel, picking up a hairbrush and combing hair, writing letters and pictures using a pencil, using chopsticks to pick up small objects, folding an umbrella, and other activities similar to those performed on a daily basis. A repetitive task practice technique typically included fist making, forearm rotation, clay squeezing and molding, griping a small ball, and pinching small coins. Although both of these techniques were always included at almost the same proportion of training time (usually 30 minutes for shaping technique and 30 minutes for repetitive task practice technique) during one-to-one training program, the program was tailored by the occupational therapist to suit the individual patient. The program was modified upon improvement of motor function of the affected upper limb, if necessary. Certain conventional approaches, such as facilitation techniques and manual dexterity exercise, were sometimes included in the program. Positive verbal guidance, encouragement and feedback were frequently applied so as to achieve the best performance for the selected tasks. The 60-min self-exercise was encouraged in another quiet room without any supervisors. Prior to each self-exercise session, the patient received written instructions prepared individually for self-exercise, of which tasks were similar to the shaping and repetitive tasks applied in the one-to-one training session, with some rest breaks of a few minutes. After each self-exercise session, the occupational therapist checked and reviewed the performance of the tasks through individual interview. The problem associated with the tasks was aggressively approached in the following one-to-one training session. At their discharge from the hospital, patients were provided with instructions prepared by the occupational therapist for daily home exercise, which were based on patients' functional status.

### Serial evaluation of motor function of the affected upper limb

The motor function of the affected upper limb was serially evaluated on the day of admission/discharge, and four weeks after discharge if possible. For the evaluation of motor function, FMA and Wolf Motor Function Test (WMFT) were administered by an occupational therapist. A number of studies investigated the psychometric properties using these two scales and reported high inter-rater and test-retest reliabilities [19-22]. FMA is a performance-based quantitative measure for the assessment of various impairments in post-stroke patients [19,20]. In FMA, the section on motor function of the upper limb consists of 33 items. As each item is rated on a three-point ordinal scale (0 = cannot, 1 = can perform partially, 2 = can perform fully), the maximum motor performance score for the upper limb that can be attained is 66 points. The WMFT contains 15 timed tasks as well as two strength tasks [21,22]. The performance time of each timed task was recorded in a single trial. When the task was not completed within 120 seconds, the performance time of the task was recorded as 120 seconds. For statistical analysis, the mean value of WMFT performance time of 15 tasks was transformed to natural logarithm to normalize the skewed distribution of the data, as applied in the analysis for EXCITE trial [12].

### Statistical analysis

For all patients, changes in two applied measures between admission and discharge were analyzed statistically. For patients in whom follow-up evaluation at four weeks after discharge was available, changes between admission and discharge and also those between admission and four weeks after discharge were analyzed. The significance of the median changes in FMA score and natural logarithm of WMFT performance time was analyzed using the signed Wilcoxon's rank sum test for paired samples. Furthermore, to identify the baseline feature that influenced the outcome of the intervention, linear regression analysis was performed. The following six baseline characteristics were selected for the analysis: age, gender, time since stroke onset, subtype of stroke, side of upper limb hemiparesis, and institution where the intervention was provided. We treated age at admission and time since stroke onset as continuous variables. Gender, subtype of stroke (intracerebral hemorrhage/cerebral cortical infarction/lacunar infarction), side of upper limb hemiparesis (dominant hand/non-dominant hand), institution where the intervention was provided, were treated as ordinal variables. Our goal was to identify factors that correlated significantly with the outcome (i.e., significant changes in two applied measures including FMA score and WMFT log performance time between admission and discharge) and then enter these factors into multivariate analysis to identify predictors of outcome. All statistical analyses were performed using The Statistical Package for Social Sciences version 17.0 (SPSS Inc., Chicago, IL). Data were expressed as mean ± SD. A *p *value less than 0.05 was considered statistically significant.

## Results

Table 2 summarizes the clinical characteristics of the studied patients. The mean age at admission was 58.5 ± 13.4 years. The period after onset of stroke ranged from 1 to 28 years (mean, 5.0 ± 4.5). The background cerebrovascular accident was intracerebral hemorrhage in 107 patients, cerebral cortical infarction in 27 and lacunar infarction in 70 patients. Follow-up evaluation at 4 weeks after discharge was performed in 79 patients (39% of all studied patients).

**Table 2:** Demographic data of the studied patients

Age at admission	
All patients, (years)	58.5 ± 13.4
< 40 (years)	19 (9)
40-50 (years)	26 (13)
50-60 (years)	59 (29)
60-70 (years)	68 (33)
≥ 70 (years)	32 (16)
Gender	
Females	73 (36)
Males	131 (64)

Time since stroke onset	
All patients, years	5.0 ± 4.5
< 2 years	53 (26)
2-5 years	76 (37)
5-10 years	54 (27)
≥ 10 years	21 (10)

Subtype of stroke	
Intracerebral hemorrhage	107 (53) (putamen: 63, thalamus: 34, brainstem: 5, subcortical: 5)
Cerebral cortical infarction	27 (13) (MCA territory: 27)
Lacunar infarction	70 (34) (CR: 26, IC: 17, BG: 17, brainstem: 10)

Side of upper limb hemiparesis	
Dominant hand	124 (61)
Non-dominant hand	80 (39)

Institution	
Jikei University Hospital	12 (6)
Jikei Daisan Hospital	56 (28)
Shimizu Hospital	98 (48)
Kenkoukai Tokyo Hospital	27 (13)
Nishi-Hiroshima Rehabilitation Hospital	11 (5)

Values are numbers (%) or mean ± standard deviation.

MCA: middle cerebral artery, BRS: Brunnstrom Recovery Stage, CR: corona radiate, IC: internal capsule, BG: basal ganglia

### Safety and feasibility of the intervention

The scheduled 15-day protocol of the intervention was completed by all 204 patients. No significant change in vital signs was observed in any patients throughout the in-patient intervention. None of the patients experienced any pathological symptoms or any deterioration of motor function in the upper limb during hospitalization. In 79 patients who received follow-up evaluation at four weeks after discharge, no new adverse symptoms or signs were recorded during the 4-week observation period after discharge.

### Changes in motor function after intervention

**Figure 2:**
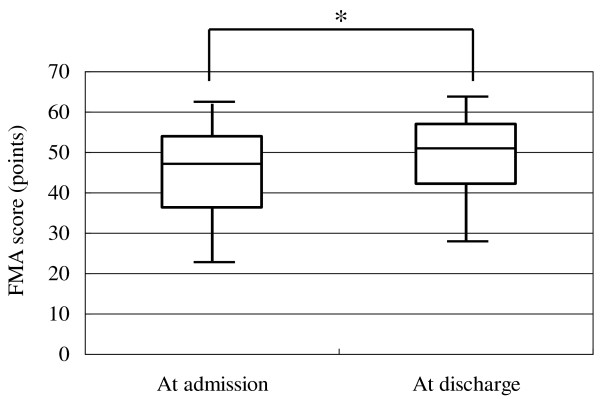
**Changes in FMA score using data of all patients**. Bars represent the median, and 5^th^, 25^th^, 75^th^, and 95^th ^percentiles. *P < 0.001.

**Figure 3:**
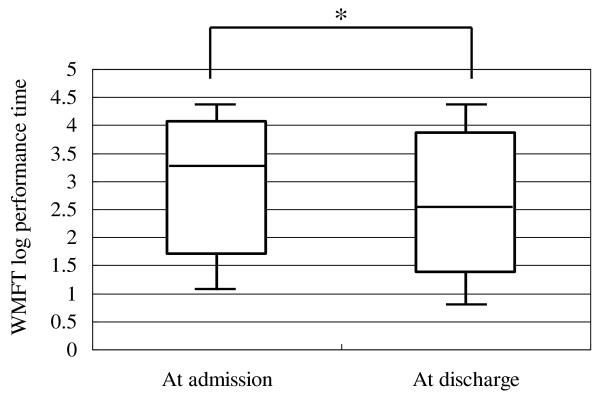
**Changes in WMFT log performance time using data of all patients**. Bars represent the median, and 5^th^, 25^th^, 75^th^, and 95^th ^percentiles. *P < 0.001.

**Figure 4:**
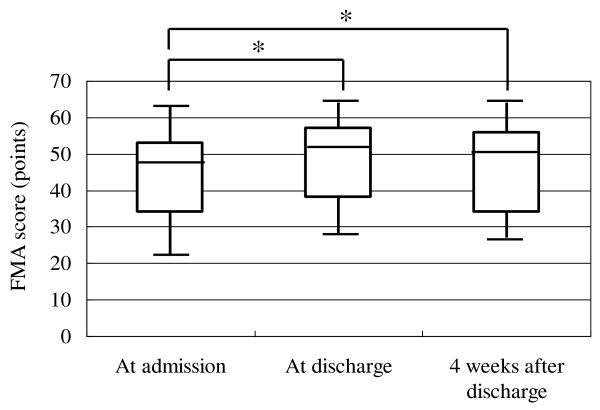
**Changes in FMA score in patients who were followed up to 4 weeks after discharge**. Bars represent the median, and 5^th^, 25^th^, 75^th^, and 95^th ^percentiles. *P < 0.001.

**Figure 5:**
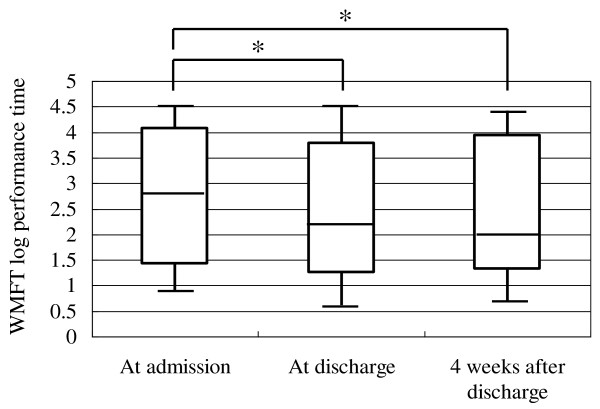
**Changes in WMFT log performance time in patients who were followed up to 4 weeks after discharge**. Bars represent the median, and 5^th^, 25^th^, 75^th^, and 95^th ^percentiles. *P < 0.001.

Analysis of data of all patients showed a mean FMA score of 44.6 points at admission and 48.6 points at discharge, and a mean WMFT log performance time of 2.93 at admission and 2.37 at discharge. Similar analysis showed a significant increase in FMA score from 47 (36-54) [median (interquartile range)] points at admission to 51 (42-57) points at discharge (p < 0.001, Figure 2). Similarly, WMFT log performance time diminished significantly after inpatient intervention in all patients from 3.23 (1.70-4.07) at admission to 2.51 (1.36-3.86) at discharge (p < 0.001, Figure 3). In 79 patients evaluated at 4 weeks after discharge, both the increase of FMA score and shortening of WMFT log performance time were significant both at discharge and at 4 weeks after discharge compared to the respective values at admission [FMA; 48 (34-53) points at admission, 51 (38-57) points at discharge, 50 (34-56) points at 4 weeks after discharge, p < 0.001, between admission and discharge, p < 0.001, between admission and 4 weeks after discharge, Figure 4. WMFT log performance time; 2.81 (1.42-4.08) at admission, 2.20 (1.25-3.78) at discharge, 2.01 (1.31-3.94) at 4 weeks after discharge, p < 0.001, between admission and discharge, p < 0.001, between admission and 4 weeks after discharge, Figure 5].

### Results of linear regression analysis

When FMA score was selected as the dependent variable, the results of the analysis found no significant relationship with any of the six parameters (Table 3). These six parameters accounted for 3.3% of the variation in FMA score. Similarly, the analysis with WMFT log-performance time as the dependent variable found no significant relationship with the same six parameters. These six parameters accounted for 2.7% of the variation in WMFT log performance time.

**Table 3:** Linear regression analysis of six baseline characteristics and changes in FMA score and WMFT log performance time.

	increase in FMA score				Shortening of WMFT log performance time			
	
	Adjusted R^2 ^= 0.033				Adjusted R^2 ^= 0.027			
	
	Β	SE	Standardized β	p value	β	SE	Standardized β	p value
Age at admission (years)	0.010	0.021	0.034	0.636	0.001	0.002	-0.008	0.912
Gender	0.043	0.597	0.005	0.943	0.044	0.068	0.047	0.523
Latency (years)	0.002	0.005	0.030	0.674	0.001	0.001	-0.045	0.530
Subtype of stroke	0.512	0.570	0.065	0.370	-0.060	0.065	-0.067	0.358
Side of hemiparesis	0.318	0.577	0.040	0.582	0.022	0.066	0.024	0.736
Institution	0.033	0.300	0.008	0.914	0.030	0.034	0.064	0.375

Latency: time between onset of stroke and intervention of magnetic stimulation and occupational therapy

## Discussion

To our knowledge, this is the first report showing the safe and feasible application of low-frequency rTMS/intensive OT across several institutions, resulting in significant improvement of motor function of the affected upper limb in post-stroke hemiparetic patients.

With regard to the safety and feasibility of the protocol, all studied patients completed the protocol without showing any adverse effects. In the original report of constraint-induced movement therapy (CIMT) program, which includes training for six hours, the protocol was considered too rigorous or intensive for some patients, because of long duration of training per day [23]. In contrast, in our protocol, the duration of training was 4 hours each day, which consisted of 2-hour one-to-one training and 2-hour self-exercise, since we expected that the brevity of training time would increase the feasibility of the protocol. The results showed none of the patient dropped out from the study complaining of the length of the training session. This shorter training time per day seemed to have provided high feasibility compared with the original CIMT program. For rTMS application, we basically followed well-established guidelines by Wassermann. Regarding the safety limitation of stimulation, Anderson et al. [24] reported that a total stimulation of less than 12,960 pulses per day and 38,880 per week can be safely tolerated in healthy volunteers. However, no researchers have investigated the safety limitation of rTMS volume (stimulation numbers) in stroke patients in chronic phases. Although the numbers of stimulation per day (2,400 pulses) and during total inpatient treatment (26,400 pulses) in our protocol were definitely higher than any other trials that investigated the therapeutic effect of rTMS for post-stroke patients, no studied patient experienced adverse effects considered to be caused by rTMS application. Our study provides one candidate value of safety limitation of rTMS volume when applied to chronic stroke patients. In this study, patients with documented epileptic discharge on pretreatment electroencephalogram or on antiepileptic medications to prevent seizure were also excluded from the study. This exclusion also may have contributed to the high safety with our proposed protocol. Therefore, we consider that our protocol is a safe intervention for post-stroke upper limb hemiparesis with high feasibility, especially in patients who meet all the inclusion criteria developed for the intervention.

Several randomized controlled trials have demonstrated the beneficial effects of low-frequency rTMS applied to the non-lesional hemisphere on motor function of the affected upper limb in poststroke patients [5-7]. Similarly, the beneficial effect of intensive OT for this patient population has been also confirmed [12]. Subsequently, we applied these two modalities simultaneously as a combination therapy for such patients. The results of this study showed that our inpatient intervention significantly improved motor function of the affected upper limb. Among the rehabilitative intervention for upper limb hemiparesis in chronic stroke, CIMT is currently considered to be most efficacious approach [25]. In the EXCITE trial which is the largest randomized controlled trial investigating the effect of CIMT, patients treated with CIMT for two weeks showed significant shortening of WMFT log performance time from 2.96 to 2.38 (the extent of decrease was 0.58) [12]. In all patients of our study, the mean WMFT log performance time decreased from 2.93 to 2.37 (the extent of decrease was 0.56). Therefore, although the inclusion criteria differed between the EXCITE trial and our study, the effect size of our protocol on motor functional recovery seems comparable to that with CIMT. Furthermore, we consider that these improvements are clinically meaningful, since all studied patients were deemed to have reached a plateau state with regard to recovery before the intervention. As mentioned above, both of these two interventions can activate local neural function in the lesional hemisphere. We speculate that the underlying mechanism of motor functional recovery in the affected upper limb is functional reorganization in the lesional hemisphere induced by both modalities. Malcolm et al. [13] introduced high-frequency rTMS combined with CIMT for treatment of post-stroke patients with upper limb hemiparesis. They applied 2000 stimulations of 20 Hz high-frequency rTMS over the lesional hemisphere and CIMT daily for two weeks, and compared the effects of the protocol on motor function of the affected upper limb with that in patients treated with CIMT only. Their results showed that although patients who received the combination protocol showed significant improvement of motor function, the extent of the improvement was not significantly different from that in patients treated with CIMT only. Based on these results, they concluded that the use of high-frequency rTMS in combination with rehabilitative training did not facilitate the functional recovery. Their study concept that rTMS application is expected to enhance motor learning in the post-stimulus period was similar to ours. The main difference between their protocol and our combination protocol was the rTMS modality; high-frequency rTMS over the lesional hemisphere in Malcolm's study vs. low-frequency rTMS over the non-lesional hemisphere in our protocol. As mentioned above, our combination protocol significantly improved motor function of the affected upper limb. However, our study was not able to investigate whether low-frequency rTMS over the non-lesional hemisphere has value as a therapeutic useful adjuvant to rehabilitative training, because of the lack of patient group for whom only rehabilitative training was provided. Further studies with patient group for comparison should be performed to address this issue. Unfortunately, we did not perform neuroimaging or neurophysiological studies to demonstrate plastic changes in the brain following the intervention. It is desired to obtain solid evidence for any functional change in the brain following rTMS/OT using investigative modalities such as functional MRI and measurement of cortical excitability with TMS. With the introduction of EEG navigated-paired pulse TMS coregistration technique, which was developed recently by Ferreri et al. [26], it may be possible to characterize the change in neural connectivity after the intervention.

Follow-up evaluation after discharge showed persistent improvement of motor function of the affected upper limb up to four weeks after discharge. Although the duration of improvement of motor function of the affected upper limb was relatively short after a single session of low-frequency rTMS [5,6], Fregni et al. [7] reported that the improvement induced by application of low-frequency rTMS to the non-lesional hemisphere daily for five consecutive days was maintained until two weeks after the intervention. In another study, the improvement of motor function of the affected upper limb in patients who received CIMT was also maintained up to several months after the intervention [12]. Whether the long-term effects of each of the two interventions are preserved remains unknown at this stage, the finding of motor function improvement at four weeks after discharge reflects the benefits of the therapeutic intervention after stroke. In this regard, it is possible that daily home exercise performed after discharge also contributed to the 4-week improvement. Further studies are needed to determine the long-term efficacy of the intervention beyond the 4-week post-discharge period.

The result of linear regression analysis showed no significant relationship between any of the six tested baseline parameters and the response to the intervention. On the basis of the findings that neither age at admission nor time after stroke influences the outcome, we speculate that the intervention can produce beneficial functional reorganization even in elderly patients and those with a long history of stroke. Consequently, one can assume that the inclusion criteria used in our protocol for optimal age and timing was significantly broad. In this study, however, no acute/subacute stroke patients within one year after onset was included based on the study inclusion criteria. It remains unknown if earlier application of the protocol during the acute/subacute phase of stroke can produce more functional improvement than those seen in our patients, since it has been reported that the beneficial functional reorganization is higher in acute/subacute phase than in later phase of stroke [27]. Furthermore, it is possible that repeated application of this 15-day inpatient protocol could result in more significant improvement of motor function, although none of the patients has so far received the 15-day protocol twice. In addition, the extent of motor functional recovery was not influenced by the institution where the protocol was applied. This means that the combination protocol developed at our department can be extended and duplicated across multiple institutions for heterogeneous population when the protocol is provided by physicians and therapists who received a training program for standardization of the protocol.

The study has few other limitations. First, the study was performed without a control group, although the number of enrolled patients was sufficiently large. To confirm the beneficial effects of our protocol, there is still a need to test the hypothesis that the intervention safely produces improvement of motor function in a randomized controlled study that includes a control group. Second, the design of this study did not allow dissection of the separate effects of each intervention; low-frequency rTMS and intensive OT, on motor function of the affected upper limb. In this regard, we view our proposed protocol of combination therapy as one entity rather than two separate interventions. However, it is important to compare the motor functional change in patients treated by the combination protocol and in those who receive only low-frequency rTMS or intensive OT. Third, the duration of rTMS application, intensive OT and in-patient treatment period were arbitrarily selected. Although the duration in the current protocol seems acceptable as mentioned above, the optimal durations need to be determined for optimal costs and recovery.

## Conclusions

Our multi-institutional study using our protocol of low-frequency rTMS and intensive OT showed significant improvement of motor function of the affected upper limb in poststroke patients. The response to the treatment was not influenced by age or time after stroke onset. Our protocol is safe, feasible, and potentially useful neurorehabilitative intervention for upper limb hemiparesis after stroke, although its efficacy should be confirmed in a randomized controlled study including a control group.

## List of abbreviations used

CIMT: constraint-induced movement therapy; FDI: first dorsal interosseous; FMA: Fugl-Meyer Assessment; MEP: motor evoked potential; OT: occupational therapy; rTMS: repetitive transcranial magnetic stimulation; WMFT: Wolf Motor Function Test.

## Competing interests

The authors declare that they have no competing interests.

## Authors' contributions

WK, MA and MU conceived of the study design, performed the data collections and processing, and drafted the manuscript. MS, JS and TO clinically applied rTMS for the patients. AY, KT, SM, HH and NU performed clinical evaluation and provided occupational therapy. All authors read and approved the final manuscript.
